# A polymorphism in the *FAM13A* gene confers protection against tuberculosis in Brazilian workers exposed to silica

**DOI:** 10.36416/1806-3756/e20240327

**Published:** 2025-05-21

**Authors:** Marcos César Santos de Castro, Angela Santos Ferreira Nani, Kaio Cezar Rodrigues Salum, Lucas de Carvalho Costa, Valéria Barbosa Moreira, Hermano Albuquerque de Castro, Patrícia Canto Ribeiro, Walter Costa, Cícero Brasileiro de Mello, Fabiana Barzotto Kohlrausch

**Affiliations:** 1. Programa de Pós-graduação em Ciências e Biotecnologia, Universidade Federal Fluminense, Niterói (RJ), Brasil.; 2. Departamento de Medicina Clínica, Hospital Universitário Antônio Pedro, Universidade Federal Fluminense, Niterói (RJ), Brasil.; 3. Universidade do Estado do Rio de Janeiro, Rio de Janeiro (RJ), Brasil.; 4. Escola Nacional de Saúde Pública, Fundação Oswaldo Cruz, Rio de Janeiro (RJ), Brasil.; 5. Departamento de Biologia Geral, Universidade Federal Fluminense, Niterói (RJ), Brasil.

**Keywords:** silicosis, tuberculosis, genetic polymorphisms, genetic association studies, cytokines

## Abstract

**Objective:**

Tuberculosis (TB) is an infectious disease caused by the bacillus Mycobacterium tuberculosis, which was recognized by the World Health Organization (WHO) as a global epidemic in 1993. TB is the leading infectious disease associated with silicosis, with studies showing an increased risk when compared to healthy individuals. We conducted an association study to evaluate the influence of polymorphisms in the ACE, FAM13A, FAS, FASLG, IL1RN, NOS2, TGFB1, and TNF genes on TB susceptibility.

**Methods:**

Nine polymorphisms were genotyped using Polymerase Chain Reaction (PCR) in a sample of 143 patients with silicosis in Rio de Janeiro (RJ), Brazil.

**Results:**

Seventy (49%) patients had a confirmed prior diagnosis of TB, of whom 25 (35.7%) had simple silicosis and 45 (64.3%) had complicated silicosis. The TG genotype of rs2609255 in FAM13A showed a protective effect against TB (OR=0.46; 95% CI: 0.22-0.98; p=0.040) compared to the GG genotype, and also when compared to the two combined homozygous genotypes (TT+GG) (OR=0.43; 95% CI: 0.20-0.90; p=0.024). Logistic regression analysis, including independent clinical variables, confirmed the protective effect of the TG genotype.

**Conclusion:**

This study suggests that the rs2609255 polymorphism in FAM13A may play a role in TB risk among patients with silicosis. Given the limited research on genetic polymorphisms and TB susceptibility in silicosis patients, further studies are needed to validate these findings.

## INTRODUCTION

Tuberculosis (TB) is an infectious disease caused by the bacillus *Mycobacterium tuberculosi*s (*M. tuberculosis*). Despite significant progress, it remains one of the most lethal infectious diseases worldwide.^1^ Several conditions increase the risk of TB, including malnutrition, alcohol abuse, smoking, anemia, diabetes, anti-TNF drugs, HIV infection, and silicosis.^2^ ^-^ ^5^

Silicosis is a fibrotic lung disease caused by the inhalation of crystalline silica particles, and TB is its most commonly associated infectious disease. Studies have shown that individuals with silicosis have a higher risk of developing TB when compared to healthy individuals.^6^ A recent meta-analysis by Ehrlich et al. (2021) further demonstrated that the severity of radiological manifestations of silicosis significantly increases the risk of TB.^6^

Tuberculosis is primarily transmitted through the air, but the mucociliary clearance mechanism plays a role in containing the bacillus.^7^ Bacilli that evade this physical barrier reach the alveolar space, triggering the intense production of reactive oxygen species (ROS) and reactive nitrogen species (RNS), which cause direct damage to invading agents.^8^ This oxidative stress environment results from increased levels of nitric oxide synthase (NOS2).^8^ ^,^ ^9^ Additionally, it stimulates the release of several interleukins, including interleukin-1 (IL-1α, IL-1β), which promote fibroblast activation and collagen fiber deposition in the alveolar space, while the IL-1 receptor antagonist (IL-1Ra) inhibits their action.^10^

Bacilli that survive this intense oxidative environment are phagocytosed by alveolar macrophages (AM), triggering various inflammatory and anti-inflammatory pathways mediated by cells and multiple cytokines.^7^ Several molecules are involved in these pathways, including tumor necrosis factor-alpha (TNFα), which is produced by activated AM and stimulates fibroblast recruitment and proliferation, in addition to serving as a ligand for apoptosis receptors.^9^ TNFα also plays a crucial role in granuloma formation, a fundamental mechanism for containing *M. tuberculosis*. Activated AM also release anti-inflammatory cytokines, such as transforming growth factor beta-1 (TGFβ1), which downregulates proinflammatory cytokines and inhibits T-cell proliferation and activation, balancing the response between bacterial eradication and host survival.^11^ Angiotensin-converting enzyme (ACE), released by epithelial cells and macrophages, is a biomarker of lung injury, and a gene polymorphism has recently been identified as a risk factor for TB.^12^ ^,^ ^13^ AM apoptosis also contributes to the pathogenesis of TB and silicosis, with increased expression of cellular apoptosis receptors (FAS) and their ligands (FASLG and TNFα), promoting the recruitment of inflammatory cells.^9^ Additionally, the interaction between AM and silica crystals elevates NOS2 levels, leading to increased nitric oxide production by macrophages and the generation of ROS and RNS, which cause extensive damage to cell membranes.^9^ ^,^ ^14^

The ability of macrophages and lymphocytes to migrate within interstitial compartments is essential for tissue surveillance and pathogen elimination. The Rho-GTPase family, a group of enzymes that bind and hydrolyze GTP, plays a fundamental role in cytoskeletal regulation and cell migration.^15^ Several pathogenic bacteria secrete proteins into host cells, acting as activators or inhibitors of Rho-GTPases, thereby facilitating their pathogenesis.^16^ *M. tuberculosis* also interferes with macrophage signaling pathways through GTP-binding proteins on Rho-GTPases, which are crucial for phagocytosis signal transduction.^16^ The FAM13A isoform 1 protein contains a Rho GTPase-activating protein (RhoGAP) domain, suggesting its involvement in modulating Rho-GTPase activity.^17^

Despite the high risk of TB, some patients with silicosis do not develop this infectious complication, suggesting that genetic polymorphisms may influence susceptibility.^8^ ^,^ ^18^ ^,^ ^19^ It is important to emphasize that silicosis is an irreversible and incurable disease, and that TB accelerates the progression of fibrosis in the lung parenchyma.^20^

Identifying biomarkers associated with a higher risk of TB in patients with silicosis is a valuable tool for disease management and follow-up. Therefore, the aim of the present study was to evaluate the influence of genetic polymorphisms in *ACE* (Ins/Del), *FAM13A* (rs2609255), *FAS* (rs2234767), *FASLG* (rs763110), *IL1RN* (rs419598 and rs2234663), *NOS2* (rs2297518), *TGFB1* (rs1800469), and *TNF* (rs1800629) on TB susceptibility in 143 Brazilian patients with silicosis. This is the first and largest Brazilian association study on genetic polymorphisms and tuberculosis in patients with silicosis.

## METHODS

### Subjects

This study included a total of 143 patients with silicosis, who were treated at the Antônio Pedro Hospital of the Federal Fluminense University, the Pedro Ernesto Hospital of the State University of Rio de Janeiro, and the National School of Public Health of the Oswaldo Cruz Foundation, all located in the State of Rio de Janeiro, Brazil. The study was approved by the Ethics Committees of these three institutions, and informed consent was obtained from all patients.

The diagnosis of silicosis was based on the occupational history of exposure to silica particles, in combination with radiological findings consistent with the disease, according to the International Classification of Radiographs of Pneumoconiosis (ILO). All chest radiographs were evaluated independently by three ILO-certified readers and classified as simple silicosis (opacities < 1.0 cm) or complicated silicosis (at least one opacity > 1.0 cm), following ILO criteria. The degree of agreement, measured using the Kappa coefficient, was 0.86. In cases of disagreement between readers regarding the classification of small and large opacities, the final result was determined by consensus among the three readers. The chest radiographs were obtained using Siemens AG equipment (model LX30; Erlangen, Germany) and a second device from VMI *Tecnologias* (LDM206-VM, MG, Brazil), applying the same radiological technique. At the time of diagnosis, all patients were removed from their workplaces. The patients included in this study were diagnosed with silicosis between 1969 and 2019, with a median diagnosis year of 2004. 

The diagnosis of TB was confirmed through the association of clinical characteristics of the infectious disease with new radiological findings, in addition to confirmatory laboratory tests, such as a positive culture for *M. tuberculosis*, adenosine deaminase (ADA) levels, polymerase chain reaction (PCR), or histopathological findings of granulomatous disease with caseous necrosis. The diagnosis of TB occurred between 1975 and 2023, with a median diagnosis year of 2005. 

### Clinical characteristics

The following social, clinical, and functional characteristics were evaluated: age (years), confirmed prior diagnosis of TB, occupational activity, silica exposure (SE; years), weekly working hours (WW; hours), total exposure time (TET; hours), exposure withdrawal (EW; years), smoking level (SL; pack-years), and use of personal protective equipment (PPE). The individual TET (in hours) was calculated by adjusting WW hours for each worked month (11/12; one-month vacation), which was then multiplied by the total number of working years. The EW (in years) was determined by subtracting the year of silicosis diagnosis (and work removal) from the year of sample collection 

### Laboratory procedures

Genetic material was collected from cell samples obtained by mouthwash with 5 mL of saline solution (NaCl 0.5%) for 60 seconds. Individual samples were identified, and genomic DNA extraction was performed as previously described.^21^

Genotyping was carried out by PCR. *IL1RN* 2018T/C (rs419598), 86 bp variable number of tandem repeats (VNTR) (rs2234663), and *ACE* Ins/Del (rs4646994) were evaluated by standard PCR, as previously described.^13^ ^,^ ^22^ ^,^ ^23^ For *ACE* rs4646994, the D allele, consisting of a 190-bp fragment, and the I allele, of a 490-bp fragment, were detected by electrophoresis of PCR products in a 2% agarose gel.

Genotypes of *IL1RN* rs2234663 (VNTR) were detected according to the PCR product sizes in a 2.5% agarose gel, which consisted of: IL1RN*1 (four repeats), 410 bp; IL1RN*2 (two repeats), 240 bp; IL1RN*3 (five repeats), 500 bp; IL1RN*4 (three repeats), 325 bp; and IL1RN*5 (six repeats), 595 bp. Restriction fragment length polymorphism (RFLP), using 1U of *Msp I* in a reaction following the manufacturer’s instructions (New England Biolabs), was used for genotyping *IL1RN* rs419598. Alleles were detected by electrophoresis in a 3% agarose gel, based on the following fragment sizes: 123 and 233 bp (C allele) and 356 bp (T allele).

 Real-time polymerase chain reaction was performed using predesigned and validated TaqMan^®^ assays (Thermo Fisher Scientific, Brazil) for genotyping *FAM13A* rs2609255 (C_15906608_10), *FAS* rs2234767 (-1377G/A; C_12123966_10), *FASLG* rs763110 (-844C/T; C_3175437_10), *NOS2* rs2297518 (Ser608Leu; C_11889257_10), *TGFB1* rs1800469 (-509C/T; C_8708473_10), and *TNF* rs1800629 (-308G/A; C_7514879_10). The samples were processed using the CFX96 real-time PCR system (Bio-Rad Laboratories, CA, USA), following the manufacturer’s instructions (Thermo Fisher Scientific, Brazil).

### Statistical analyses

Gene counting was performed to analyze allele frequencies. Deviations from Hardy-Weinberg equilibrium were evaluated using the χ^2^ test. The χ^2^ test or Fisher’s exact test was also used to perform association analyses between gene polymorphisms and TB. The t-test and Mann-Whitney test were used to analyze quantitative clinical characteristics with normal distribution and those with deviations from normal distribution, respectively, as determined by the Kolmogorov-Smirnov test. Values were presented as mean ± standard deviation (SD). 

The odds ratio (OR) and 95% confidence interval (95% CI) for independent predictors of TB susceptibility were evaluated using multivariate logistic regression analysis. The logistic regression model included the following independent variables: polymorphisms significantly associated with TB, TET > 44,229 hours, EW in years, and silicosis severity (complicated silicosis). Statistical tests were performed using SPSS version 20.0 software (SPSS Inc., Chicago, IL, USA). A p-value < 0.05 was considered significant.

## RESULTS

### Clinical and radiological characteristics

Among the 143 patients, 66 (46%) worked in sandblasting, the most prevalent occupation in our sample. Other occupations included marble working (21 patients, 15%), hammer drilling (18 patients, 12%), civil construction (17 patients, 12%), and various other activities (tilemaker, sandblasting manager, welder, painter, ceramicist), which accounted for 21 patients (15%).

Seventy patients (49%) had a confirmed prior diagnosis of TB, including 25 (35.7%) with simple silicosis and 45 (64.3%) with complicated silicosis (p=0.321). Among these 70 TB cases, 57 (81%) had pulmonary tuberculosis, 11 (16%) had pleural tuberculosis, and 2 (3%) had lymph node tuberculosis. The clinical parameters and diagnostic procedures are shown in Table 1. 

**Table 1:** Procedures and methods for the diagnosis of tuberculosis.

TUBERCULOSIS	PULMONARY	PLEURAL	LYMPH NODE	TOTAL
	57 (81%)	11 (16%)	2 (3%)	70
DIAGNOSTIC PROCEDURE				
SPONTANEOUS SPUTUM	38	0	0	38
INDUCED SPUTUM	03	0	0	03
BRONCHOSCOPY / BAL	16	0	0	16
THORACOCENTESIS (WITH PLEURAL BIOPSY)	0	11	0	11
LYMPH NODE BIOPSY	0	0	02	02
TOTAL				70
DIAGNOSTIC METHOD				
ACID-FAST BACILLI (AFB) TEST	39	0	0	39
MYCOBACTERIAL CULTURE (WITH OR WITHOUT POSITIVE AFB)	45	0	02	47
MOLECULAR TESTING - PCR (*M. tuberculosis*)	12	0	0	12
ADA (ADENOSINE DEAMINASE)	0	11	0	11
HISTOPATHOLOGY (GRANULOMA WITH CASEOUS NECROSIS)	2	9	02	13

BAL, bronchoalveolar lavage; PCR: polymerase chain reaction.

Regarding the radiographic classification of patients without a confirmed prior diagnosis of TB according to the ILO 2022 criteria, 28 patients were categorized as having simple chronic silicosis, with categories 1 (1/1 and 1/2) and 2 (2/1 and 2/2) being the most prevalent in this group, accounting for 68% of cases. In this group, 37 patients presented with large opacities and were classified as follows: 13 (35%) as category A, 17 (46%) as category B, and 7 (19%) as category C. Among patients with a confirmed prior diagnosis of TB, as classified by the ILO 2022, 25 were categorized as having simple chronic silicosis, with categories 2 (2/1 and 2/2) and 3 (3/2 to 3/3) being the most prevalent, together comprising 73% of cases. In this group, 42 patients presented with large opacities and were classified as follows: 11 (26%) as category A, 19 (45%) as category B, and 12 (29%) as category C. Other clinical and demographic characteristics are presented in Tables 2 and 3. 

**Table 2:** Distribution of sociodemographic and clinical characteristics between patients with and without a confirmed prior diagnosis of tuberculosis.

Variables	Total (143)	No tuberculosis (73)	Tuberculosis (70)	p
Age (years)	59.98±8.81	60.60±8.99	59.34±8.63	0.395^a^
SE (years)	21.55±9.31	21.49±10.02	21.61±8.58	0.865^b^
WW (hours)	47.06±9.37	47.53±9.32	46.57±9.46	0.759^b^
TET (hours)	44,229±19,939	44,771±21,100	43,663±18,788	0.741^a^
EW (years)	14.00±10.19	13.75±9.88	14.23±10.55	0.880^b^
SL (pack-years)	36.15±30.52	36.05±25.69	36.26±34.62	0.404^b^

SE, silica exposure in years; WW, weekly working hours; TET, total exposure time (in hours); EW, exposure withdrawal (in years); SL, smoking level (in pack-years). Values expressed as mean ± standard deviation. ^a^t-test, Kolmogorov-Smirnov: p>0.05; bMann-Whitney test, Kolmogorov-Smirnov: p<0.05

**Table 3:** Silicosis - clinical classification.

CLINICAL CLASSIFICATION	WITHOUT TUBERCULOSIS	WITH TUBERCULOSIS	TOTAL
ACUTE	0 (%)	0 (%)	0 (0%)
SUBACUTE PROGRESSIVE (ACCELERATED SILICOSIS)	8 (73%)	3 (27%)	11 (8%)
CHRONIC	65 (49%)	67 (51%)	132 (92%)
CLASSIFICATION: CHRONIC SILICOSIS			
CHRONIC SIMPLE	28 (53%)	25 (47%)	53 (40%)
CHRONIC COMPLICATED	37 (47%)	42 (53%)	79 (60%)

No statistically significant differences were observed in silica exposure (SE) (p=0.865), weekly working hours (WW) (p=0.759), total exposure time (TET, hours) (p=0.741), exposure withdrawal (EW) (p=0.880), or smoking level (SL) (p=0.404) between patients with and without a confirmed prior diagnosis of TB. 

### Association analyses

The genotype distribution of *ACE* (Ins/Del), *FAM13A* (rs2609255), *FAS* (rs2234767), *FASLG* (rs763110), *IL1RN* (rs419598 and rs2234663), *NOS2* (rs2297518), *TGFB1* (rs1800469), and *TNF* (rs1800629) polymorphisms was in equilibrium according to the Hardy-Weinberg principle. 

A summary of genotype and allele distribution in the total sample and among individuals with a confirmed prior diagnosis of TB is provided in Table 4. A statistically significant association was observed between rs2609255 in *FAM13A* and TB. The TG genotype exhibited a protective effect against TB (OR=0.46; 95% CI: 0.22-0.98; p=0.040). Additionally, the analysis revealed that the *FAM13A* TG genotype was also a predictor of protection against TB (OR=0.43; 95% CI: 0.20-0.90; p=0.024) when compared to the combined homozygous genotypes (TT+GG) (Table 4). No significant associations were found for the remaining polymorphisms.

**Table 4:** Genotype and allele distribution in patients with and without a history of tuberculosis.

Polymorphism	Total	No tuberculosis	Tuberculosis	p	OR (95%CI)
*ACE* rs4646994	121	61	60		
DD	72	37 (0.61)	35 (0.58)	0.942	1.00
DI	45	22 (0.36)	23 (0.38)		1.11 (0.52-2.33)
II	4	2 (0.03)	2 (0.03)		1.06 (0.07-15.31)
D	189	96 (0.79)	93 (0.78)	0.823	1.00
I	53	26 (0.21)	27 (0.23)		1.07 (0.58-1.97)
*FAM13A* rs2609255	132	67	65		
TT	79	36 (0.54)	43 (0.66)	0.040	1.00
TG	45	29 (0.43)	16 (0.25)		0.46 (0.22-0.98)
GG	8	2 (0.03)	6 (0.09)		2.51 (0.41-26.67)
T	203	101 (0.75)	102 (0.78)	0.552	1.00
G	61	33 (0.25)	28 (0.22)		0.84 (0.47-1.49)
TT+GG	87	38 (0.57)	49 (0.75)	0.024	1.00
TG	45	29 (0.43)	16 (0.25)		0.43 (0.20-0.90)
*FAS* rs2234767	129	67	62		
GG	97	52 (0.78)	45 (0.73)	0.709	1.00
GA	32	15 (0.22)	17 (0.27)		1.17 (0.51-2.66)
G	226	119 (0.89)	107 (0.86)	0.540	1.00
A	32	15 (0.11)	17 (0.14)		1.26 (0.60-2.65)
*FASLG* rs763110	97	52	45		
CC	30	18 (0.35)	12 (0.27)		1.00
CT	39	21 (0.40)	18 (0.40)	0.584	1.29 (0.49-3.37)
TT	28	13 (0.25)	15 (0.33)		1.73 (0.61-4.91)
C	99	57 (0.55)	42 (0.47)	0.258	1.00
T	95	47 (0.45)	48 (0.53)		1.39 (0.79-2.44)
*IL1RN r*s419598	143	73	70		
TT	92	45 (0.62)	47 (0.67)	0.498	1.00
TC	49	26 (0.36)	23 (0.33)		0.85 (0.42-1.70)
CC	2	2 (0.03)	0 (0.00)		0.00 (0.00-5.31)
T	233	116 (0.79)	117 (0.84)	0.370	1.00
C	53	30 (0.21)	23 (0.16)		0.76 (0.42-1.39)
*IL1RN* rs2234663	141	72	69		
11	107	55 (0.76)	52 (0.75)	0.844	1.00
12	26	12 (0.17)	14 (0.20)		1.23 (0.52-2.91)
13	4	3 (0.04)	1 (0.01)		0.35 (0.01-4.59)
22	4	2 (0.04)	2 (0.04)		1.06 (0.07-15.08)
1	244	125 (0.87)	119 (0.86)	0.679	1.00
2	34	16 (0.11)	18 (0.13)		1.18 (0.58-2.42)
3	4	2 (0.01)	1 (0.00)		0.35 (0.01-4.45)
*NOS2* rs2297518	132	54	78		
GG	94	50 (0.70)	44 (0.72)	0.144	1.00
GA	35	21 (0.30)	14 (0.23)		0.76 (0.34-1.67)
AA	3	0 (0)	3 (0.05)		
G	223	121 (0.85)	102 (0.84)	0.720	1.00
A	41	21 (0.15)	20 (0.16)		1.13 (0.58-2.20)
*TGFB1* rs1800469	139	71	68		
CC	63	32 (0.45)	31 (0.46)	0.992	1.00
CT	55	28 (0.39)	27 (0.40)		1.00 (0.49-2.07)
TT	21	11 (0.15)	10 (0.15)		0.94 (0.35-2.52)
C	181	92 (0.65)	89 (0.65)	0.909	1.00
T	97	50 (0.35)	47 (0.35)		0.97 (0.59-1.59)
*TNF* rs1800629	140	72	68		
GG	120	62 (0.86)	58 (0.85)	0.890	1.00
GA	20	10 (0.14)	10 (0.15)		1.07 (0.41-2.64)
G	260	134 (0.93)	126 (0.93)	0.894	1.00
A	20	10 (0.07)	10 (0.07)		1.06 (0.43-2.64)

The multiple logistic regression model, including the potential independent risk variables for TB susceptibility, is presented in Table 5. After adjusting for other risk factors, rs2609255 in *FAM13A* was the only independent variable significantly associated with TB. The *FAM13A* TG genotype exhibited a protective effect against TB susceptibility (OR=0.32; 95% CI: 0.13-0.75; p=0.009), while no effect was observed for EW, TET, or silicosis severity (complicated silicosis).

**Table 5:** Multivariate logistic regression model for independent predictors of tuberculosis susceptibility.

Variables	B	SE	Wald	df	p	OR	95% CI
*FAM13A* (TG)	-1.15	0.44	6.84	1	0.009	0.32	0.13-0.75
TET > 44,229 (hours)	0.16	0.42	0.14	1	0.704	1.17	0.51-2.68
Complicated silicosis	0.04	0.42	0.01	1	0.920	1.04	0.46-2.37
Exposure withdrawal (years)	0.001	0.02	0.001	1	0.948	1.00	0.96-1.04

Legend: B, estimated coefficient; SE, standard error; Wald, test for the statistical significance of each coefficient (B) in the model (*Z* statistic); df, degree of freedom; OR, odds ratio; CI, confidence interval.

## DISCUSSION

The respiratory system has mechanisms that prevent the entry of various agents, such as particulate inorganic material, viruses, bacteria, and mycobacteria. Additionally, several leukocytes that compose the immune system contribute to the defense process, with AM being the most important.^24^ ^,^ ^25^

Macrophages phagocytose bacilli and, after breaking them down, present their main antigens to lymphocytes. This antigen presentation triggers a cascade of biological events in lymphocytes, including activation, clonal expansion, and cytokine secretion, which in turn activate other macrophages. AM also serve as a reservoir for harboring bacilli, allowing intracellular multiplication. The bacilli are ultimately eliminated from the AM interior through apoptosis.^26^

The ability of leukocytes to migrate through interstitial compartments is essential for tissue surveillance and pathogen elimination. Each stage of this migration relies on the dynamic regulation of the cytoskeleton. The Rho-GTPase families play a fundamental role in cytoskeleton regulation, thereby ensuring adequate cell migration.^15^ Rho-GTPases transmit signals from cell surface receptors and regulate the actin cytoskeleton and microtubules, controlling cell shape, polarity, mobility, and adhesion.^17^

Changes in Rho-GTPases have been described in lung diseases such as asthma, COPD, and acute lung injury,^17^ and are also associated with infectious diseases.^16^ *Salmonella typhimurium*, for example, secretes the SptP protein, which functions as a GTPase-activating protein (GAP) on Rho-GTPases, influencing the host cell.^27^

Tuberculosis is also associated with Rho-GTPases.^16^ Sun et al. (2010) described how the mycobacterial nucleoside diphosphate kinase protein (Ndk) exhibited activity on the GTPase-activating protein, inhibiting phagosome maturation and thereby promoting the survival of mycobacteria within macrophages.^28^ The same authors also demonstrated that the NdK protein was associated with a reduction in nitric oxide production mediated by NOS2, interfering with apoptosis dependent on reactive oxygen species and affecting the activity of Rho-GTPases.^29^

The FAM13A isoform 1 protein contains a RhoGAP domain, suggesting its involvement in modulating the activity of Rho-GTPases.^17^ The FAM13A (family with sequence similarity 13, member A) is expressed in the airways, particularly in type II epithelial cells and macrophages of human lungs, and its coding gene is located on chromosome 4q22.^17^ ^,^ ^30^ ^,^ ^31^ Two variants of *FAM13A* have been identified in human lung tissue, with 24 exons and 17 exons, respectively.^17^ Corvol et al. (2014) described how genetic variation in *FAM13A* affects Rho-GTPase activation and is associated with cellular pathways, though its exact biological function remains poorly understood.^17^

In this study, we observed an association between the rs2609255 polymorphism of the *FAM13A* gene and TB in patients with silicosis. The TG genotype showed a protective effect against TB susceptibility in these patients. Since the *FAM13A* protein contains a RhoGAP domain, the rs2609255 polymorphism in *FAM13A* could act as a modulator of Rho-GTPase activity, thereby promoting host protection mechanisms against *Mycobacterium tuberculosis* bacilli. However, no functional study of this polymorphism has been conducted to date. Other studies have also shown associations between polymorphisms in *FAM13A* and various lung diseases, such as idiopathic pulmonary fibrosis,^31^ ^,^ ^32^ asthma,^33^ chronic obstructive pulmonary disease (COPD),^34^ cystic fibrosis,^35^ and also increased susceptibility to silicosis.^30^ This increased susceptibility to silicosis could be explained by other mechanisms. A recent study revealed that *FAM13A* can activate the Wnt pathway, a network of protein interactions that regulates several cellular functions, including cell differentiation, migration, and proliferation, and controls the protein stability of β-catenin.^36^ The Wnt/β-catenin pathway has been reported to play a role in the development and progression of silicosis, with β-catenin acting as a key regulator of this process.^37^ ^,^ ^38^ Moreover, the Wnt/β-catenin pathway has been shown to be significantly activated in lung tissue from patients with lung fibrosis, and the aberrant activation of this pathway may induce increased fibroblast migration.^39^ In addition, the pharmacological and genetic inhibition of the Wnt /β-catenin pathway may attenuate, or even reverse, lung fibrosis.^40^

No associations were observed with the other polymorphisms evaluated in this study. This lack of association may be attributed to certain study limitations, such as the inability to analyze all pathways of TB pathogenesis and the small sample size. Further studies are needed to investigate the associations between genetic polymorphisms and the pathophysiological mechanisms of TB in patients with silicosis. Given the limited number of studies correlating genetic polymorphisms with TB susceptibility in patients with silicosis, additional research is required to confirm our findings.
